# Structural and spectroscopic analyses of the sporulation killing factor biosynthetic enzyme SkfB, a bacterial AdoMet radical sactisynthase

**DOI:** 10.1074/jbc.RA118.005369

**Published:** 2018-09-14

**Authors:** Tsehai A. J. Grell, William M. Kincannon, Nathan A. Bruender, Elizabeth J. Blaesi, Carsten Krebs, Vahe Bandarian, Catherine L. Drennan

**Affiliations:** From the Departments of ‡ Chemistry and; ** Biology and; ‡‡ Howard Hughes Medical Institute, Massachusetts Institute of Technology, Cambridge, Massachusetts 02139,; § Department of Chemistry, University of Utah, Salt Lake City, Utah 84112, and; Departments of ¶ Chemistry and; ‖ Biochemistry and Molecular Biology, The Pennsylvania State University, University Park, Pennsylvania 16802

**Keywords:** enzyme catalysis, enzyme structure, metalloenzyme, crystal structure, catalysis, AdoMet radical enzymes, iron sulfur clusters, radical SAM, sactionine linkage, sporulation killing factor

## Abstract

Sactipeptides are a subclass of ribosomally synthesized and post-translationally modified peptides (RiPPs). They contain a unique thioether bond, referred to as a sactionine linkage, between the sulfur atom of a cysteine residue and the α-carbon of an acceptor residue. These linkages are formed via radical chemistry and are essential for the spermicidal, antifungal, and antibacterial properties of sactipeptides. Enzymes that form these linkages, called sactisynthases, are AdoMet radical enzymes in the SPASM/Twitch subgroup whose structures are incompletely characterized. Here, we present the X-ray crystal structure to 1.29-Å resolution and Mössbauer analysis of SkfB, a sactisynthase from *Bacillus subtilis* involved in making sporulation killing factor (SKF). We found that SkfB is a modular enzyme with an N-terminal peptide-binding domain comprising a RiPP recognition element (RRE), a middle domain that forms a classic AdoMet radical partial (β/α)_6_ barrel structure and displays AdoMet bound to the [4Fe-4S] cluster, and a C-terminal region characteristic of the so-called Twitch domain housing an auxiliary iron-sulfur cluster. Notably, both crystallography and Mössbauer analyses suggest that SkfB can bind a [2Fe-2S] cluster at the auxiliary cluster site, which has been observed only once before in a SPASM/Twitch auxiliary cluster site in the structure of another AdoMet radical enzyme, the pyrroloquinoline quinone biosynthesis enzyme PqqE. Taken together, our findings indicate that SkfB from *B. subtilis* represents a unique enzyme containing several structural features observed in other AdoMet radical enzymes.

## Introduction

**Figure 1.**
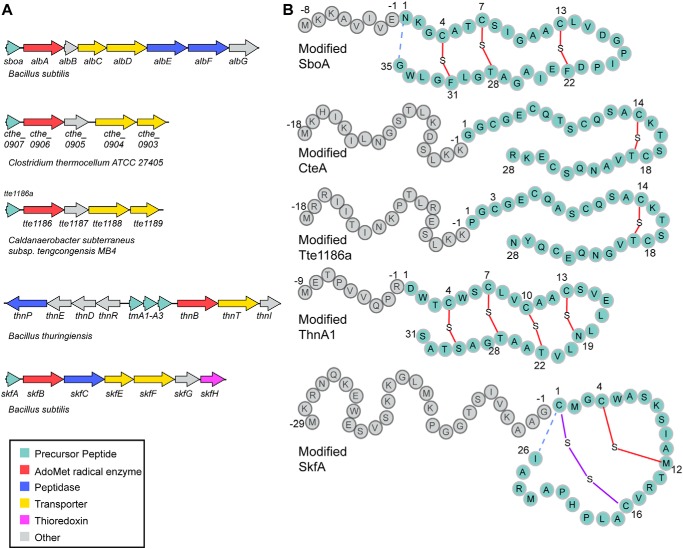
**Sactipeptide biosynthesis.** *A*, gene scheme of several RiPP biosynthetic gene clusters involved in the biosynthesis of sactipeptides. These biosynthetic clusters encode precursor peptides (*cyan*); AdoMet radical enzymes (*red*), which are responsible for installing the unique sactionine linkages characteristic of the sactipeptide natural products; peptidases (*blue*), which are responsible for cleaving the leader peptide; and ABC transporters, which are involved in transporting the sactipeptide out of the producing organism. *B*, the precursor peptides for each biosynthetic gene cluster in *A* are shown with the leader peptide sequence, which binds to the biosynthetic enzymes, in *gray* and the mature peptide, which undergoes post-translational modifications, in *cyan*. The sactionine linkages formed between the sulfur atom of a cysteine residue and the α-carbon of an acceptor residue are shown in *red*. Final maturation steps of SboA and SkfA involve an N- to C-terminal macrocyclization (*dashed blue lines*) step concurrent with a leader peptide cleavage step. Maturation of SkfA also includes disulfide bond formation (*purple*).

Sulfur to alpha-carbon cross-linked peptides (sactipeptides)^5^ are a subclass of ribosomally synthesized and post-translationally modified peptide (RiPP) natural products characterized by the presence of one or more thioether bonds between the sulfur atom of a cysteine residue and the α-carbon of an acceptor residue, forming a so-called sactionine linkage (see Fig. 1) (1). To date, six sactipeptides have been identified, and most show chemical and metabolic stability and a narrow spectrum of antimicrobial activity (2–6).

Biosynthesis of sactipeptides commences with a leader peptide–dependent post-translational modification of the precursor peptide core by colocalized radical enzymes, herein called sactisynthases (see Fig. 1*A*) (1, 2, 7–10). Known sactisynthases include AlbA, SkfB, and presumably CteB, which install a sactionine linkage on precursor peptides SboA, SkfA, and CteA, respectively, during the maturation of the sactipeptides subtilosin A (8), sporulation killing factor (7), and thermocellin (11), respectively (see Fig. 1). These sactisynthases are members of the *S*-adenosyl-l-methionine (AdoMet) radical enzyme superfamily, which utilize a canonical C*X*_3_C*X*φC motif (where φ is a conserved aromatic amino acid) to coordinate a [4Fe-4S] cluster, thereby named the AdoMet radical cluster (12). AdoMet binds to the site-differentiated iron of the AdoMet radical cluster and is reductively cleaved to produce a highly reactive 5′-deoxyadenosyl radical (5′-dAdo^•^) species. 5′-dAdo^•^ subsequently abstracts a hydrogen atom from the substrate, forming 5′-deoxyadenosine and a substrate radical, which undergoes further transformation to form the desired product. In the case of the characterized sactisynthases, H-atom abstraction has been shown to occur from the α-carbon of the acceptor residue (8, 13–15).

In addition to a characteristic AdoMet radical core, which houses the active site, sactisynthases are predicted to contain an N-terminal peptide-binding domain called a RiPP recognition element (RRE) (16) and a C-terminal SPASM or Twitch domain (17). RREs are expected to bind the leader sequences of the peptide substrates to secure the substrate to the enzyme, whereas the SPASM or Twitch domains are expected to coordinate additional or auxiliary iron-sulfur clusters (Aux I and Aux II), whose function in sactipeptide generation is not fully established.

SPASM and Twitch domains are not unique to sactisynthases. They are found in several thousand AdoMet radical enzymes that display activities ranging from the dehydrogenation reaction catalyzed by butirosin biosynthetic enzyme BtrN to the amino acid cross-linking reaction catalyzed by the streptide-like biosynthetic enzyme SuiB (Fig. S1). SPASM domains are characterized by the presence of a conserved seven-cysteine motif (C*X*_9–15_G*X*_4_C_gap_C*X*_2_C*X*_5_C*X*_3_C_gap_C) that is used to coordinate two iron-sulfur clusters, Aux I and Aux II (17, 18). The name “SPASM” is derived from the biosynthetic products of the first set of enzymes identified: AlbA, PqqE, anaerobic sulfatase maturating enzyme (anSME), and MftC, which biosynthesize subtilosin A, pyrroloquinoline quinone, anaerobic sulfatase, and mycofactocin, respectively. The so-called Twitch domain is a truncated version of the SPASM domain that binds only one auxiliary cluster (19).

The first set of structures of SPASM and Twitch domain–containing enzymes showed variations in the cysteine ligation of the Aux clusters. anSME (SPASM subclass) and BtrN (Twitch subclass) displayed Aux clusters that were fully ligated, whereas the molybdenum cofactor biosynthetic enzyme MoaA displayed an open coordination site for the binding and positioning of the substrate GTP in the active site (19–21). For anSME and BtrN, auxiliary clusters have been suggested to play a role in oxidizing the radical intermediate species to produce product as neither of these enzymes use their cluster for substrate binding (20). The only structure of a sactisynthase determined to date, CteB, revealed a SPASM domain with two auxiliary clusters and an open coordination site on Aux I (11), the cluster closest to the active site. However, instead of binding the cysteine residue that forms the C–S bond linkage, as predicted for the AlbA system (8), a cysteine residue from the leader sequence of substrate CteA is coordinated to Aux I. The 21-mer CteA fragment utilized in crystallography includes the leader peptide sequence (residues −1 to −18) and the first three residues of the core peptide but did not contain the cysteine involved in sactionine formation (11) (see Fig. 1*B*). Thus, the role of the Aux I cluster in CteB is not clear at this time.

Here, we present the first view of the sactisynthase SkfB, a Twitch domain–containing family member. SkfB initiates thioether cross-link formation between the α-carbon of Met^12^ of the precursor peptide SkfA (Fig. 1*B*) during the maturation of the sactipeptide sporulation killing factor in *Bacillus subtilis* (1, 7, 13). Although the role of the Aux cluster in SkfB is unknown, it is required for overall activity (7), and recent studies utilizing a radical clock substrate where Met^12^ is replaced by a cyclopropylglycine residue show that ring opening occurs in a variant where the Aux cluster has been removed (15). The cyclopropylglycine-containing substrate does not undergo thioether cross-link formation. These studies suggest that in SkfB, Aux I does not play any role in the initial H-atom transfer events, but rather in the late stages of the functionalization of the peptide. To further probe the role and nature of Aux I in SkfB, here we investigated SkfB both by crystallography and spectroscopy.

## Results

### Overall architecture of SkfB is modular

**Figure 2.**
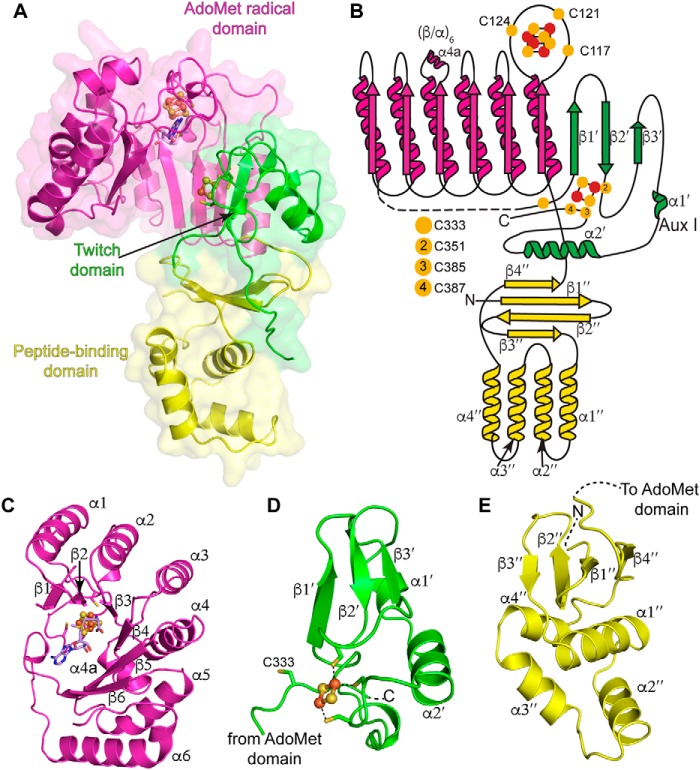
**Overall architecture of SkfB.** *A*, the partial (β/α)_6_ TIM barrel of the AdoMet radical domain (*magenta*) houses the [4Fe-4S] AdoMet radical cluster and an AdoMet molecule (*lilac*). The C-terminal domain adopts a Twitch domain architecture (*green*), providing three visible ligands to a [2Fe-2S] auxiliary cluster, Aux I. The N-terminal peptide-binding domain (*yellow*) displays the architecture of the RRE motif identified in peptide-binding domains of RiPP biosynthetic enzymes. *B*, topology diagram of SkfB colored by domain with the AdoMet radical domain in *magenta*, the Twitch domain in *green*, and the RRE domain in *yellow. Yellow circles* indicate cysteine residues, and *orange* and *yellow circles* represent the iron and sulfur atoms, respectively, of the clusters shown as *ball* and *stick* representations. *C–E* show a closer look at the individual domains of SkfB. *C*, the active site of SkfB is located within the inner cavity of the AdoMet radical domain and comprises six parallel β-strands. The binding sites of the AdoMet radical cluster and AdoMet (*lilac*) are located at the top of the partial barrel. *D*, the C-terminal Twitch domain (*green*) contains the canonical elements of a SPASM/Twitch fold, a β-hairpin, β1′ and β2′, followed by α2′. In SkfB, a short β-strand, β3′, and α-helix α1′ are found in between β2′ and α2′. *E*, the N-terminal peptide-binding domain of SkfB (*yellow*) folds into a three-stranded antiparallel β-sheet (β1″–β3″) and a consecutive helical bundle (α1″–α4″), reminiscent of the winged helix turn helix motif that comprises the RRE. The N-terminal domain ends with a parallel β-strand, β4″ adjacent to β1″. The clusters are shown as *ball* and *stick* representations with iron atoms colored *orange* and sulfur atoms colored *yellow*.

The structure of SkfB from *B. subtilis* in complex with AdoMet was solved to 1.29-Å resolution using an iron single-wavelength anomalous dispersion technique (Table 1). The final structure contained one molecule in the asymmetric unit with residues 12–322 and 330–401, a molecule of AdoMet bound to a [4Fe-4S] AdoMet radical cluster, and a [2Fe-2S] auxiliary cluster. The SkfB architecture is modular, folding into three distinct domains: an N-terminal peptide-binding domain, an AdoMet radical domain, and a C-terminal Twitch domain (Fig. 2).

**Table 1:** **Data collection and refinement statistics for SkfB structure** The highest-resolution shell is shown in parentheses. r.m.s., root mean square; asu, asymmetric unit.

	SkfB	
	Iron peak*^a^*	Remote
**Data collection**		
Beamline	Home Source	APS 24-ID-C
Wavelength (Å)	1.5418	0.9792
Space group	*C*2	*C*2
Cell dimensions (Å)	76.5, 83.1, 61.3	76.5, 83.1, 61.3
Resolution (Å)	50.0–1.97 (2.00–1.97)	50.0–1.29 (1.34–1.29)
Unique reflections	52,216	92,230
Completeness	97.8 (69.0)	96.5 (76.2)
Redundancy	3.6 (2.4)	5.6 (2.6)
*R*_sym_*^b^*	0.043 (0.119)	0.037 (0.499)
*I*/σ(*I*)	29.5 (7.7)	43.6 (1.8)
CC1/2*^d^*		(0.729)

**Model refinement**		
Resolution limits (Å)		50.0–1.29
*R*_work_/*R*_free_*^c^*		14.25/16.85
No. molecules in asu		1
No. atoms		
Protein		3,226
[Fe-S]		12
AdoMet		27
Water		390
*B*-factors (Å^2^)		
Protein		20.2
[Fe-S]		15.8
AdoMet		13.74
Water		30.7
r.m.s. deviations		
Bond lengths (Å)		0.006
Bond Angles (°)		0.927
Rotamer outliers (%)		0.87
Ramachandran plot (%)		
Most favored		98.15
Additionally allowed		1.85
Disallowed		0.0

*^a^* Bijvoet pairs were scaled separately in this data set.

*^b^ R*_sym_ = Σ*_hkl_*Σ*_i_* |*I*_*i*_^*hkl*^ − 〈*I^hkl^*〉|/Σ*_hkl_*Σ*_i_I*_*i*_^*hkl*^.

*^c^ R*-factor = Σ(|*F*_obs_| − *k*|*F*_calc_|)/Σ(|*F*_obs_|) and *R*_free_ of the *R* value for a test set of reflections consisting of 7.5% of the diffraction data not used in refinement.

*^d^* CC1/2 is Pearson's correlation coefficient.

### SkfB adopts a canonical AdoMet radical domain

**Figure 3.**
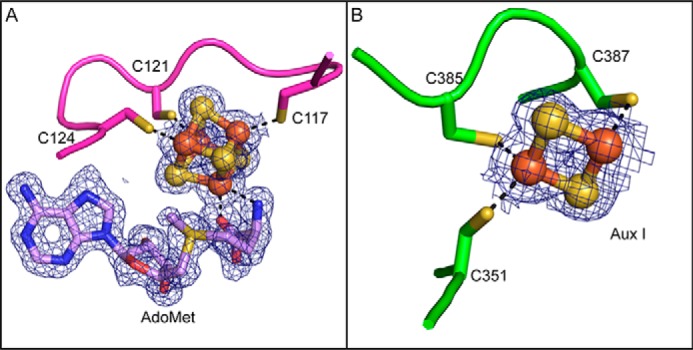
**Iron-sulfur clusters and AdoMet density.** *A*, the characteristic C*X*_3_C*X*φC motif, Cys^117^, Cys^121^, and Cys^124^, coordinates the AdoMet radical cluster in SkfB. An AdoMet molecule ligates the site-differentiated iron through a bidentate interaction with the nitrogen of the α-amino moiety and oxygen from the α-carboxyl moiety. *B*, a [2Fe-2S] cluster is observed in the Aux I site of the Twitch domain, bound by a C*X*_33_C*X*C sequence, Cys^351^, Cys^385^, and Cys^387^. 2*F_o_* − *F_c_* composite omit density is shown in *blue* and contoured at 1σ.

The central domain of SkfB (residues 106–322) folds into a partial (β/α)_6_ TIM barrel with six parallel β-strands comprising the inner face of the barrel and six α-helices flanking the outside of the barrel, providing flexibility to the rigid barrel architecture (Fig. 2, *A–C*). This architecture is characteristic of AdoMet radical enzymes and provides a binding site for cofactors and substrates in a protected cavity that facilitates the corresponding radical mechanisms of members of the superfamily. Herein, we will refer to the partial (β/α)_6_ TIM barrel architecture as the AdoMet radical domain. Residues from the canonical C*X*_3_C*X*φC motif, Cys^117^, Cys^121^, and Cys^124^, are located on the loop following β1 and ligate an essential [4Fe-4S] cluster, the AdoMet radical cluster, leaving a unique iron for direct coordination of AdoMet (Figs. 2, *A–C*, and 3*A*).

**Figure 4.**
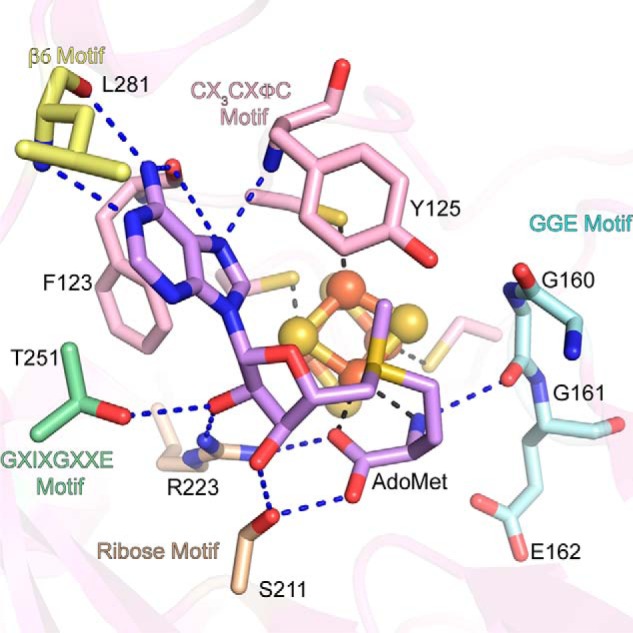
**AdoMet-binding motifs are conserved in SkfB.** The AdoMet-binding pocket is located within the partial TIM barrel (*translucent pink*) and includes interactions from the GGE motif, Gly^160^, Gly^161^, and Glu^162^ (*cyan*); the ribose motif, Ser^211^ and Arg^223^ (*tan*); G*X*I*X*G*XX*E or β5 motif, Thr^251^ (*green*); and β6 motif (*yellow*), Leu^281^. Arg^223^ plays a dual role, positioning both the carboxyl group and the ribose ring. Hydrophobic interactions from the C*X*_3_C*X*φC motif Phe^123^, and the adjacent residue, Tyr^125^ (*pink*), orient the adenine ring of AdoMet (*lilac*) in the active site. The AdoMet radical cluster is shown as a *ball* and *stick* representation with iron atoms colored *orange* and sulfur atoms colored *yellow*.

In the structure of SkfB, the α-amino and α-carboxyl groups of AdoMet are found 2.3 and 2.2 Å, respectively, from the site-differentiated iron of the AdoMet radical cluster. Conserved interactions from the AdoMet radical domain help position AdoMet in a catalytically competent orientation in the active site with all known motifs represented (Fig. 4) (22, 23). In particular, hydrogen bonds from the conserved GGE motif, Gly^160^, Gly^161^, and Glu^162^, and Arg^223^ orient the α-amino and α-carboxyl moieties of AdoMet. The ribose motif, Ser^211^, and residue Arg^223^, located adjacent to helix α4a, serve to position the hydroxyls of the ribose ring in the active site. The adenine moiety of AdoMet is oriented in the active site through hydrophobic interactions from Thr^251^ of the “G*X*I*X*G*XX*E” (or β5 motif) and from Phe^123^ of the C*X*_3_C*X*φC motif. Polar backbone interactions from the β6 motif, Leu^258^, and from the hydrophobic residues of the C*X*_3_CXφC motif, Phe^123^ and Tyr^125^ (Fig. 4), further help to orient AdoMet in the active site.

### The Twitch domain binds a [2Fe-2S] auxiliary cluster

**Figure 5.**
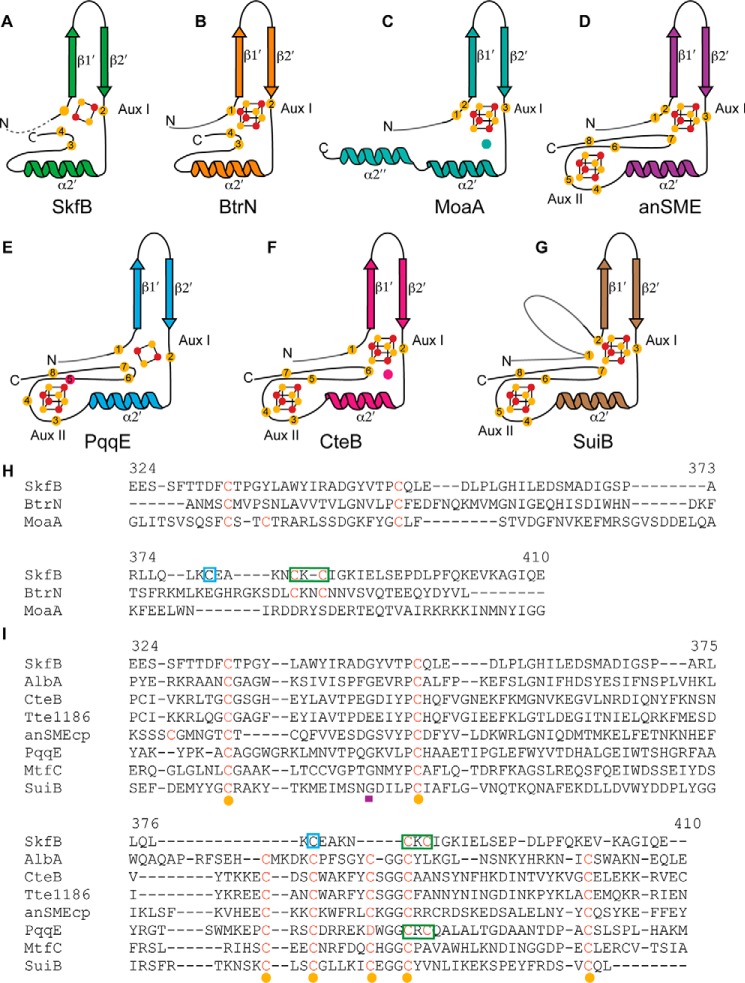
**Comparison of auxiliary cluster binding by SPASM/Twitch domains.** The canonical structural elements of the Twitch **(***A–C***)** and SPASM (*D–F*) domain are shown for each enzyme. *A*, SkfB (*green*) binds a [2Fe-2S] cluster using one cysteine following the β-hairpin and two cysteines following α2′, similar to BtrN. SkfB appears to have an open coordination site: although the cysteine corresponding to the cluster ligand before the β-hairpin motif is present, this cysteine is ∼12 Å away from the cluster. *B*, the Twitch domain of BtrN (*orange*) provides four cysteine residues to fully ligate a [4Fe-4S] auxiliary cluster. *C*, MoaA (*teal*) uses a Twitch domain to bind a [4Fe-4S] cluster with an open coordination site. The unique iron is ligated by substrate, GTP, (*teal circle*). *D*, anSME (*purple*) utilizes a SPASM motif to bind two fully ligated [4Fe-4S] clusters, Aux I and Aux II. *E*, PqqE (*blue*) binds two fully ligated clusters, a [2Fe-2S] Aux I cluster and a [4Fe-4S] Aux II cluster, using a unique aspartic acid (*red circle*) ligand. *F*, CteB (*pink*) also binds two [4Fe-4S] auxiliary clusters with an open coordination site on Aux I to which a cysteine residue from the substrate, CteA (*pink circle*), is bound in the structure. *G*, the SPASM domain of SuiB ligates two auxiliary clusters. The first cysteine ligand to Aux I is provided by the linker region connecting the SPASM domain to the AdoMet radical domain. The cluster-binding cysteines are shown as *yellow circles*, and the cluster iron and sulfur atoms are represented as *orange* and *yellow circles*, respectively. The linker regions are denoted in *gray. H*, Twitch domain sequence alignment. Residue numbers are for SkfB. Cysteine ligands to Aux I are indicated in *red*, *yellow spheres* represent the first four cysteines of the seven-cysteine motif (C*X*_9–15_G*X*_4_C_gap_C*X*_2_C*X*_5_C*X*_3_C_gap_C), and a *purple rectangle* denotes the conserved glycine residue. The C*X*C of SkfB is indicated by a *green box*, and a *blue box* designates the cysteine previously proposed to bind the Aux I cluster of SkfB (C*X*_4_C*X*C) (7). *I*, sequence alignments of SPASM domains with the sequence of SkfB (sequence numbers indicated for SkfB). Cluster-coordinating cysteines are denoted in *red*, cysteines corresponding to the seven-cysteine motif (C*X*_9–15_G*X*_4_C_gap_C*X*_2_C*X*_5_C*X*_3_C_gap_C) are indicated by *yellow circles*, and the conserved glycine residue of this motif is denoted by a *purple rectangle. Green boxes* denote the C*X*C of SkfB and PqqE, and the *blue box* indicates the cysteine previously proposed to bind the Aux I cluster of SkfB (C*X*_4_C*X*C) (7).

Following helix α6, the SkfB structure wraps back to the N terminus of the AdoMet radical domain and places β1′ of the C-terminal domain adjacent to β1, extending the inner parallel β-sheet by one strand (Fig. 2*B*). The majority of the linker between the AdoMet radical domain and the C-terminal domain (residues 322–329) is disordered in SkfB. Following the first visible residue of the C-terminal region (Thr^330^), the structure folds into a three-stranded antiparallel β-sheet, β1′–β3′, followed by two α-helices, a short α1′ and a longer α2′ (Fig. 2, *A*, *B*, and *D*). This C-terminal domain ligates Aux I, which in this structure is a [2Fe-2S] cluster (Fig. 3*B*). Cluster ligation involves one cysteine from the loop following β2′, Cys^351^, and two cysteines from the loop following α2′, Cys^385^ and Cys^387^ (Fig. 2*B*). This architecture is as expected for a Twitch domain (Fig. 5, *A–D*) (17, 19, 20) and is highly similar to the first half of a SPASM domain (Fig. 5, *A* and *E–G*), but the identity of the auxiliary cluster as a [2Fe-2S] cluster was a surprise. Although a [2Fe-2S] cluster is observed in the Aux I–binding site of PqqE (Fig. 5*E*), all other structurally characterized SPASM- or Twitch-containing AdoMet radical enzymes have Aux I clusters that are [4Fe-4S] clusters (Table S1) (11, 19, 20, 24–26).

**Figure 6.**
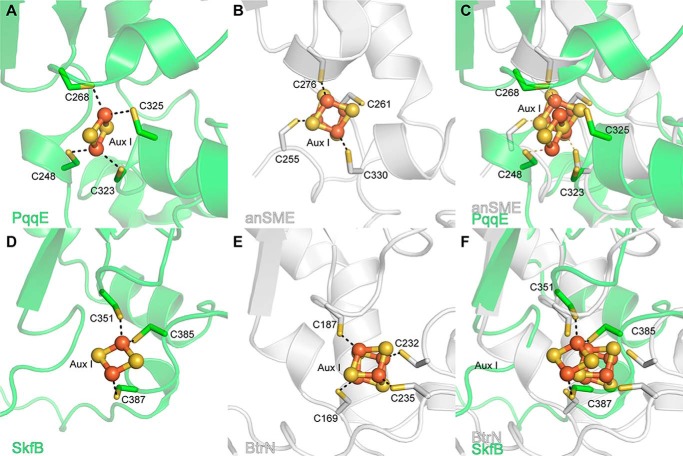
**Comparison of Aux I iron-sulfur clusters in SPASM/Twitch AdoMet radical enzymes.** *A*, the Aux I site of the PqqE SPASM domain (*green*) has been shown biochemically to bind either a [4Fe-4S] cluster or a [2Fe-2S] cluster. In the recent structure of PqqE (PDB code 6C8V), the Aux I cluster-binding site is populated by a [2Fe-2S] cluster with two of the ligands provided by a C*X*C motif, Cys^323^ and Cys^325^. *B*, anSME (PDB code 4K37), shown in *white*, contains a [4Fe-4S] cluster at the Aux I cluster-binding site ligated by Cys^255^, Cys^261^, Cys^276^, and Cys^330^. *C*, when overlaid with anSME (*white*), the cysteine positions of the PqqE (*green*) active site appear to be amenable for binding a [4Fe-4S] cluster. *D*, the Twitch Aux I site of SkfB (*green*) can bind a [2Fe-2S] cluster with cysteines from the C*X*C motif, Cys^385^ and Cys^387^, binding different irons of the cluster. *E*, the Twitch Aux I site of BtrN (PDB code 4M7T), shown in *white*, fully ligates a [4Fe-4S] auxiliary cluster. *F*, the Aux I site of SkfB (*green*) overlays well with the Aux I site BtrN (*white*). To allow for [4Fe-4S] cluster binding, the loop containing residues of the C*X*C motif of SkfB would have to move slightly. This rearrangement appears possible as this loop corresponds to the C-terminal end of the structure and is highly flexible.

Both SkfB and PqqE utilize a C*X*C sequence, Cys^385^ and Cys^387^ and Cys^323^ and Cys^325^, respectively, following helix α2 to ligate the visualized [2Fe-2S] cluster where each cysteine coordinates a different iron of the cluster. A C*X*C cluster-binding sequence has not been seen in the other SPASM/Twitch enzymes structurally characterized (Figs. 5, *H* and *I*, and 6). When the Aux I sites of SkfB and PqqE are overlaid with a [4Fe-4S]-containing Twitch and SPASM enzyme, respectively, the observed [2Fe-2S] cluster superimposes well with Aux I [4Fe-4S] clusters (Fig. 6), and that a [4Fe-4S] cluster should be able to be accommodated at this site as well (Fig. 6).

In terms of cluster ligation, the SPASM enzymes anSME, SuiB, and PqqE and the Twitch enzyme BtrN have fully ligated auxiliary clusters (Fig. 5), whereas the Twitch enzyme MoaA and the SPASM enzyme CteB have an open coordination site on Aux I. The current structure of SkfB reveals an open coordination on Aux I, which is surprising as the sequence of SkfB predicts five cysteines in the C-terminal region, four of which are conserved (Fig. 5*H*). The fourth conserved cysteine, Cys^333^, was expected to ligate the cluster from its position in the sequence before the β-hairpin in SkfB; coordination of the cluster by a cysteine in this position has been observed in all other characterized SPASM/Twitch family members. However, in this structure, Cys^333^ is visible about 12 Å away from the cluster on the linker region that connects the AdoMet domain to the Twitch domain (Fig. S2).

### Mössbauer spectroscopy

**Figure 7.**
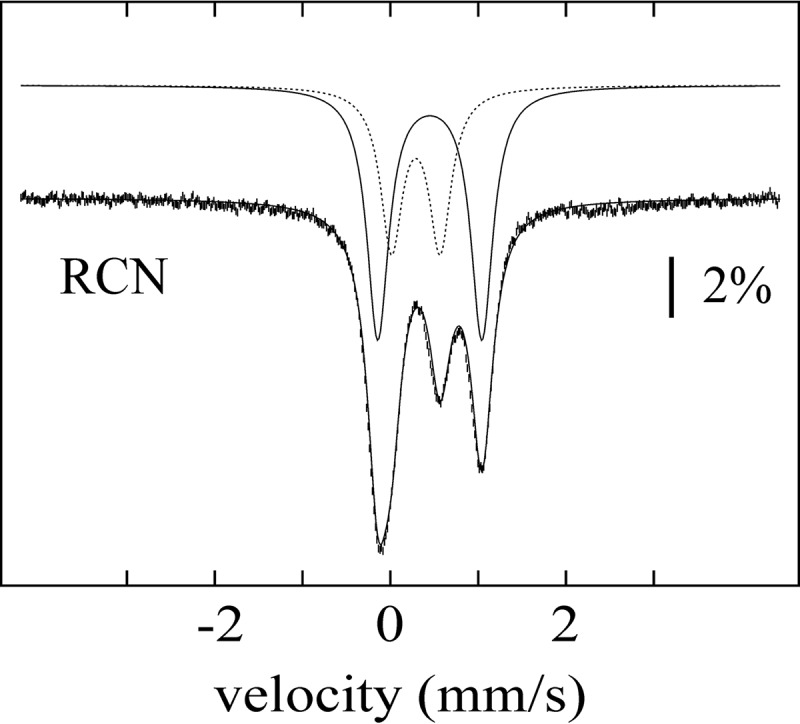
**Mössbauer spectrum of SkfB.** The spectrum of a sample of SkfB reconstituted with ^57^Fe (*vertical bars*) was recorded with the sample kept at 4.2 K in a 53-mT magnetic field applied parallel to the γ-beam. The *solid line* overlaid with the data is a simulation with two quadrupole doublets using parameters quoted in the text. The individual contributions of the quadrupole doublets associated with the [2Fe-2S]^2+^ and [4Fe-4S]^2+^ clusters are shown as *dashed* and *solid lines* above the data, respectively. *RCN*, reconstituted.

The surprising observation of a [2Fe-2S] cluster in the Aux I site was independently verified by ^57^Fe Mössbauer spectroscopy. The 4.2 K, 53-mT Mössbauer spectrum of reconstituted SkfB (Fig. 7, *vertical bars*) can be simulated with two quadrupole doublets with parameters typical of [2Fe-2S]^2+^ clusters (isomer shift δ = 0.29 mm/s and quadrupole splitting parameter Δ*E_Q_* = 0.55 mm/s; 37% of total intensity, *dotted line* above the data) and [4Fe-4S]^2+^ clusters (δ = 0.44 mm/s and Δ*E_Q_* = 1.18 mm/s; 59% of total intensity, *solid line* above the data). Together with the ^57^Fe/SkfB stoichiometry of 5.41 (determined by ICP-MS), these results indicate the presence of 1.0 [2Fe-2S]^2+^ and 0.8 [4Fe-4S]^2+^ clusters per polypeptide, consistent with the notion that SkfB harbors one [2Fe-2S] and one [4Fe-4S] cluster. Addition of the cosubstrate, AdoMet, to the sample leads to a noticeable broadening of the high-energy line of the [4Fe-4S]^2+^ cluster (Fig. S3). The observed perturbation is consistent with binding of AdoMet to the unique iron site of the [4Fe-4S]^2+^ cluster and can be rationalized with a site-differentiated mixed-valent (Fe_2_)^5+^ pair as was observed for other AdoMet radical enzymes previously (27–29).

We further investigated a reconstituted sample of the SkfB variant in which the three cysteines coordinating the AdoMet radical cluster were substituted with alanine residues (ΔRS SkfB). The Mössbauer spectrum of this variant (Fig. S4) exhibits only one quadrupole doublet with parameters virtually identical to those of the [2Fe-2S]^2+^ cluster observed in WT SkfB, consistent with notion that the [2Fe-2S]^2+^ cluster resides in the Aux I site and that the AdoMet radical cluster is a [4Fe-4S]^2+^ cluster.

### The N-terminal peptide-binding domain of SkfB contains a RiPP recognition element motif

The N-terminal domain of SkfB (residues 12–105) sits below the N-terminal region of the AdoMet domain and the C-terminal Twitch domain (Fig. 2, *A* and *B*). It folds first into a three-stranded antiparallel β-sheet, β1″–β3″, followed by a helical bundle comprising four consecutive α-helices, α1″–α4″, that stacks against the lower face of the antiparallel β-sheet (Fig. 2*E*). Following α4″, SkfB folds into a fourth β-strand, β4″, before connecting to the AdoMet radical domain. It is the upper face of the antiparallel β-sheet that forms the interface with the AdoMet radical domain and Twitch domain.

**Figure 8.**
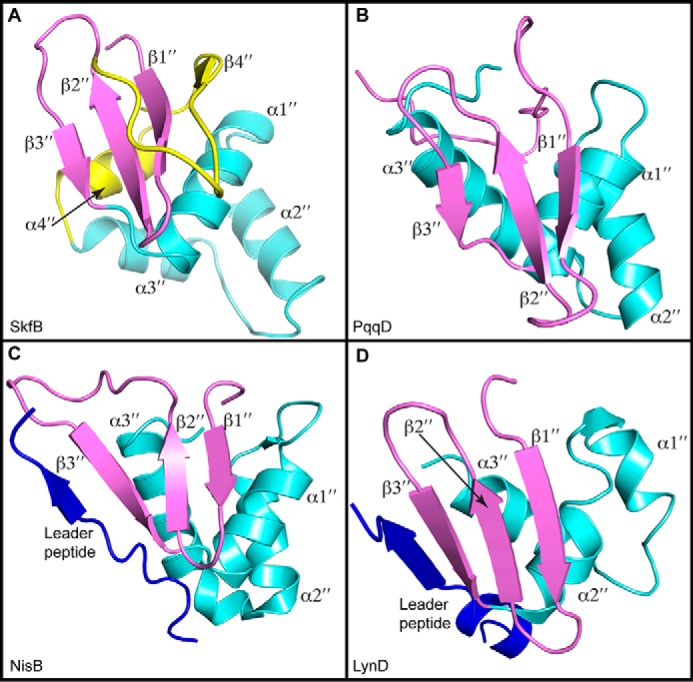
**Structural comparisons of RRE domains.** *A*, SkfB exhibits a canonical RRE comprising a three-strand antiparallel β-sheet (β1″–β3″ in *purple*) and three consecutive α-helices (α1″–α3″ in *cyan*). Following the RRE motif, SkfB folds into an additional α-helix, α4″, and β-strand, β4″ (*yellow*). *B*, the small peptide-binding protein PqqD (PDB code 5SXY) is a standalone RRE domain. *C*, the RRE domain of NisB (PDB code 4WD9) binds the leader peptide sequence of the peptide substrate NisA (*blue*) by extending the antiparallel β-sheet or the wing. *D*, the leader peptide of PatE (*blue*) also binds to the RRE domain of LynD (PDB code 4V1T) through interactions with the wing.

The N-terminal domain architecture of SkfB (Figs. 2*E* and 8*A*) is reminiscent of a peptide-binding protein that is involved in the biosynthesis of pyrroloquinoline quinone, PqqD (Fig. 8*B*), the peptide-binding domains of a nisin biosynthetic enzyme, NisB (Fig. 8*C*), and a cyanobactin biosynthetic enzyme, LynD (Fig. 8*D*), an architecture termed the RRE (16, 30–33). The RRE is responsible for binding the substrate precursor peptide in RiPP biosynthetic enzymes and folds into a winged helix-turn-helix motif composed of a three-stranded antiparallel β-sheet and three α-helices. As visualized in NisB and LynD, the leader sequence of the precursor peptide binds to the RRE motif by forming a β-strand, extending the antiparallel β-sheet or the “wing” (Fig. 8, *C* and *D*). In SkfB, the antiparallel β-sheet, β1″–β3″, and α1″–α3″ constitute the RRE motif (Figs. 2, *B* and *E*, and 8*A*).

### Structural comparison of AdoMet radical RiPP biosynthetic enzymes

**Figure 9.**
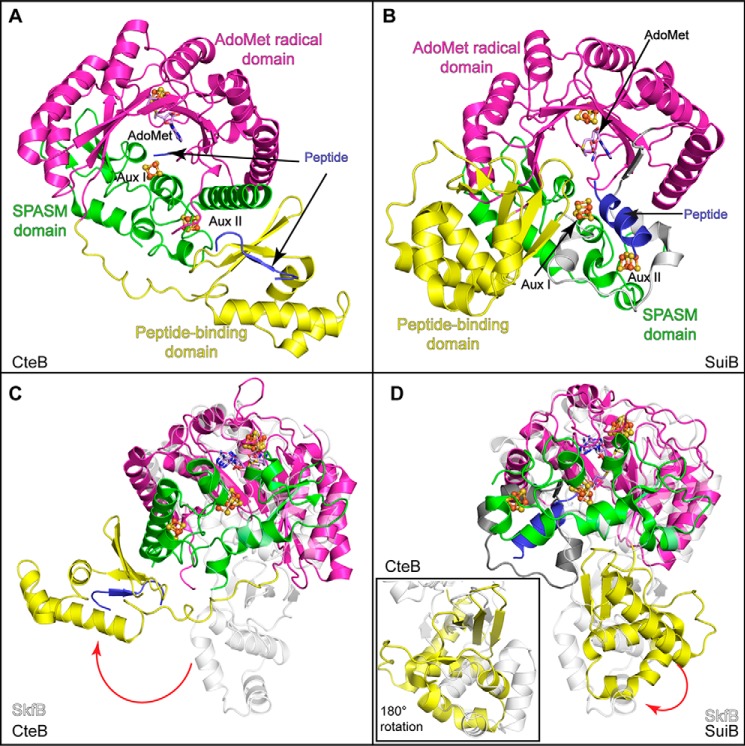
**Structural comparisons of SkfB with other AdoMet radical enzymes involved in RIPP biosynthesis.** *A*, CteB (PDB code 5WGG) exhibits a trimodular fold composed of a peptide-binding or RRE domain (*yellow*) followed by an AdoMet-binding domain (*magenta*), which binds the AdoMet radical cluster and AdoMet (*lilac*). The C-terminal end of CteB binds two clusters, Aux I and Aux II, using the SPASM domain architecture (*green*). The leader sequence of CteA (*blue*) binds to the RRE domain by extending the antiparallel β-sheet. *B*, SuiB (PDB code 5V1T) demonstrates a similar modular fold to CteB (*A*). Interestingly, the substrate for SuiB, SuiA (*blue*), is observed making contacts with the insertion (*gray*) between the AdoMet radical domain (*magenta*) and the SPASM domain (*green*) and not with the peptide-binding domain (*yellow*). *C*, the AdoMet radical and Twitch domains of SkfB (*white*) overlay well with CteB, but the peptide-binding domains are located on opposite sides of the AdoMet radical domain. *D*, SkfB (*white*) overlays well with SuiB, and the positions of the N-terminal domains show modest differences. A view of the overlaid N-terminal domains SkfB and SuiB, rotated 180°, is shown in the *inset*.

In addition to SkfB, the structures of two other AdoMet radical enzymes involved in the biosynthesis of RiPPs have been determined: the sactisynthase CteB (Fig. 9*A*) and the enzyme SuiB (Fig. 9*B*), which is involved in cross-linking two amino acid side chains (Fig. S1). Similar to SkfB, both CteB and SuiB are members of the SPASM/Twitch subfamily and adopt a modular overall architecture comprising three domains: an N-terminal peptide-binding RRE domain, a central AdoMet radical domain, and a C-terminal SPASM domain in place of the C-terminal Twitch domain visualized in SkfB. CteB and SuiB structures contain three [4Fe-4S] clusters, including the AdoMet radical cluster, bound by the canonical C*X*_3_C*X*φC motif, and two auxiliary clusters, Aux I and Aux II, bound by the SPASM domain. In addition, the linkers between the AdoMet radical domain and the SPASM domains were visualized in these structures. In SuiB, this linker region donates the first cysteine ligand to Aux I, resulting in two fully ligated auxiliary clusters (Fig. 5*G*). In CteB, Aux I has an open coordination site, which is ligated by a cysteine from the N terminus of the peptide substrate CteA (Fig. 5*F*). Structural comparisons show that the AdoMet radical domain and SPASM domains of CteB and SuiB overlay well with the AdoMet radical and Twitch domains of the SkfB structure (Fig. 9, *C* and *D*). The main differences between SkfB and CteB or SuiB are that SkfB has a Twitch rather than SPASM domain, the ligation and nature of the Aux I cluster, and the location of the N-terminal peptide-binding domain.

The N-terminal domains of both CteB and SuiB adopt typical RRE folds. As aforementioned for NisB and LynD, the leader peptide, CteA, adopts a β-strand conformation and interacts with β3″ of CteB, extending the antiparallel β-sheet of the RRE domain (Fig. 9*A*). Surprisingly, the leader peptide of SuiA binds to SuiB without interacting with its RRE domain. Instead, SuiA interacts with the AdoMet radical domain, the SPASM domain, and the linker between these two domains (Fig. 9*B*). There is considerable variability in the placement of the RRE domain with respect to the other domains. CteB has a long flexible linker between α3″ of the RRE domain and β1 of the AdoMet radical domain (Fig. 9*A*), which could allow flexibility in the positioning of the RRE domain with respect to the rest of the protein structure. In fact, the RRE domain of CteB and the RRE domain of SkfB are observed on the opposite sides of the AdoMet radical domain (Fig. 9*C*). The placement of the RRE domain of SuiB is more similar to that observed in SkfB (Fig. 9*D*). Shorter linkers between the RRE domain and the AdoMet radical domain are observed in SuiB (five residues) and SkfB (nine residues) than is seen in CteA (22 residues).

## Discussion

Here, we present the structure of an AdoMet radical Twitch enzyme, SkfB, involved in maturation of a sactipeptide natural product. The structure of SkfB allows us to compare and contrast structural features with the previously solved SPASM domain–containing RiPP biosynthetic enzymes, SuiB and CteB, as well as other SPASM and Twitch domain–containing AdoMet radical enzymes for which structures have been determined, anSME, BtrN, MoaA, and PqqE.

In terms of the overall structure, SkfB adopts a modular architecture that resembles SuiB and CteB. The Twitch domain of SkfB overlays well with the SPASM domains of CteB and SuiB but ligates one auxiliary cluster, Aux I, instead of two, a noted difference between SPASM and Twitch enzymes. Surprisingly, the SkfB structure revealed a [2Fe-2S] Aux I cluster ligated by a C*X*_33_C*X*C sequence. This cluster identity was unexpected as the initial biochemical and spectroscopic characterization proposed that SkfB contained a [4Fe-4S] auxiliary cluster ligated by a C*X*_4_C*X*C sequence (7). Moreover, most of the SPASM/Twitch enzymes characterized to date contain [4Fe-4S] auxiliary clusters. However, Mössbauer analysis on the samples prepared in a similar manner as those used for crystallographic analysis also shows that the predominant forms of the clusters in SkfB are a [4Fe-4S] cluster and a [2Fe-2S] with the spectral feature of the [4Fe-4S] cluster perturbed by AdoMet binding. This result is consistent with the AdoMet radical cluster being the one [4Fe-4S] cluster present in this sample.

The recent structure of the SPASM enzyme PqqE revealed a [2Fe-2S] cluster in the Aux I site, which has been reported to be able to bind both [4Fe-4S] and [2Fe-2S] clusters (26, 34, 35). Interestingly, like SkfB, the two-cysteine ligands to Aux I after helix α2′ are provided by a C*X*C sequence, a motif found primarily in the coordination of [2Fe-2S] clusters (36, 37). Because of the proximity of the cysteine ligands in C*X*C sequences, both cysteines are expected to ligate the same iron of the [2Fe-2S] clusters. This is not the case in SkfB or PqqE: the C*X*C sequence ligates a different iron atom of the [2Fe-2S] cluster. This coordination geometry could also allow for [4Fe-4S] cluster binding in SkfB and PqqE with minimal perturbations of the Aux I site as in both cases the site appears to be amenable to rearrangement. The malleability of this site could explain why different cluster identities have been observed in the biochemical and structural data. It is possible that the C*X*C sequence might be indicative of a physiologically relevant [2Fe-2S] cluster, or it could make a [4Fe-4S] cluster more susceptible to degradation. To understand which species is catalytically relevant, enzyme activity needs to be correlated to cluster content.

The coordination state of the auxiliary clusters of members of the SPASM and Twitch family has long been of debate (11, 17, 19, 20, 38). Although both anSME and BtrN structures reveal fully ligated clusters, an insufficient number of cysteines in the C-terminal sequence of AlbA (seven cysteines to ligate two auxiliary clusters) led to the proposition that AlbA and other sactisynthases may utilize an open coordination site for substrate ligation. It was also hypothesized that these enzymes could utilize a noncysteine ligand as has been seen in LipA (Ser) (39, 40), BioB (Arg) (41), and recently PqqE (Asp) as a ligand to the Aux II cluster (26, 38). In contrast, the C-terminal Twitch domain of SkfB contains five conserved cysteines, four of which correspond with conserved auxiliary cluster ligands in both Twitch and SPASM domains. Therefore, the structures of both SPASM and Twitch sactisynthases were highly anticipated in hope they would shed light on the cluster coordination conundrum.

The first structure of a sactisynthase, CteB, revealed that there is an open coordination site on Aux I of the SPASM domain, whereas Aux II is fully ligated (11). Interestingly, extra density inconsistent with a protein residue was observed near the site-differentiated iron of Aux I and was attributed to a cysteine residue (Cys^3^ of CteA in Fig. 1*B*) from the N-terminal fragment of CteA cocrystallized with CteB. The N-terminal fragment used in cocrystallization encompassed the leader peptide sequence (residues −18 to −1) and the first three residues of the core peptide (residues 1–3) of CteA (Fig. 1*B* and Table S1) but did not include the cysteine involved in thioether bond formation. This cysteine ligation to Aux I could foreshadow binding of the reactive cysteine (Cys^14^) in the presence of full-length CteA.

The structure of SkfB shows an open coordination site on Aux I. This finding was unexpected as C-terminal sequence alignment of SkfB with SPASM/Twitch enzymes predicts four conserved cysteine ligands (Fig. 5, *H* and *I*). Interestingly, the fourth cysteine residue, Cys^333^, was observed in the structure a distance away from Aux I. Because of the disorder in this region, however, we cannot rule out that Cys^333^ could serve as a cluster ligand in some enzyme states. A structure of SkfB in which the linker is ordered will be important for making a final determination. Regardless, the structure of SkfB leaves open the possibility that substrate will interact directly with Aux I. Recent studies (15) suggest that Aux I is not essential for binding substrate as hydrogen atom abstraction from substrate can occur without Aux I, indicating that substrate binds and is positioned correctly for the AdoMet radical chemistry in the absence of this cluster. It was also shown that the Aux I is essential for later steps in catalysis, suggesting that interaction between the substrate and Aux I could be important for cyclization, cysteine deprotonation, oxidation of a product-based radical species to generate product, or possibly all three.

The observation that Aux I is not essential for substrate binding (15) is consistent with the presence of a peptide-binding RRE domain as part of the modular structure of SkfB. The three RiPP biosynthetic AdoMet radical enzymes structurally characterized to date all have similar RRE domains, but these domains are present in three different orientations. In the structures of SkfB and SuiB, the RRE domain sits below the AdoMet radical core and SPASM/Twitch domain, whereas the RRE domain of CteB interacts with helix α6 of CteB on the top side of the AdoMet radical core. Mobility of the RRE domain might be important for positioning substrate into the active site, and the structures of SkfB, CteB, and SuiB considered here could represent snapshots of those movements rather than representing enzyme-specific variations.

We predict that SkfA will bind to SkfB using the leader peptide sequence to extend the antiparallel β-sheet of the RRE as seen with CteB. However, the distance between the RRE domain and the active site (indicated open coordination site on Aux I) is short, about 20 Å, compared with 33 Å in CteB. To position the 55-mer SkfA precursor peptide in a catalytically competent orientation, it is possible that SkfA will adopt an ordered secondary structure, the RRE domain of SkfB will undergo a conformational change upon SkfA binding, or a combination of both. How the full-length precursor peptides bind to sactisynthases to allow for sactionine linkage formation and possible conformational changes associated with this interaction still remains to be determined.

**Figure 10.**
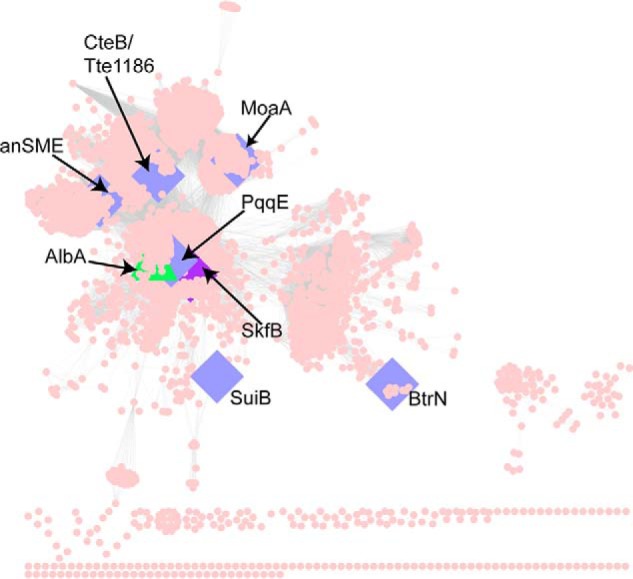
**SPASM/Twitch subclass sequence similarity network.** The protein similarity network (51) for the SPASM/Twitch subclass is visualized in Cytoscape (52) at a blast probability of 10^−20^. Sequences were obtained from the Structure Function Linkage Database (53) (http://sfld.rbvi.ucsf.edu/django; Please note that the JBC is not responsible for the long-term archiving and maintenance of this site or any other third party-hosted site.), and each node represents sequences that share 50% identity. Nodes corresponding to the previously solved members of the SPASM/Twitch subclass are represented as *blue diamonds* (11, 19, 20, 24–26), and *green* indicates family members that have been biochemically characterized but not structurally characterized (8). SkfB, which is structurally characterized here, is shown as a *purple diamond*.

Overall, SkfB represents a unique hybrid of features observed in other AdoMet radical enzymes. It has the same domain organization as CteB and SuiB but has a Twitch, not a SPASM, domain. SkfB's Twitch domain is like that of BtrN and MoaA, but its Aux I cluster is most like the Aux I cluster found in PqqE and not in BtrN or MoaA. A sequence similarity network (Fig. 10) shows that SkfB and PqqE are close in sequence space despite one having a Twitch domain and the other having a SPASM domain. Twitch domain–containing enzymes (MoaA, BtrN, and SkfB) do not cluster together nor do SPASM-containing enzymes (anSME, CteB, SuiB, and PqqE). Clustering is not based on function either, with dehydrogenases BtrN and anSME on opposite sides of the Cytoscape diagram (Fig. 10). Thus, it is difficult to predict fold or function based on sequence similarity networks for this AdoMet radical enzyme subfamily. This modular collection of domains seems well-suited to carry out a variety of chemical reactions, and with sections of sequence space left uncharacterized by crystallography, more mix-and-match combinations of domains and cluster types may be left to discover.

## Experimental procedures

### Cloning of SkfB and preparation of constructs to express variants

The *skfB* gene was previously amplified from the *B. subtilis* subsp. *subtilis* strain 168 genome and cloned into a pET28a vector containing a tobacco etch virus cleavable N-terminal His_6_ tag (13). The ΔRS SkfB construct, which encodes a C117A/C121A/C124A triple variant, was generated by site-directed mutagenesis using the primers listed in Table S2. Standard Sanger sequencing at the University of Michigan DNA Sequencing Core confirmed the sequence of each construct.

### Purification of SkfB

WT and ΔRS SkfB were purified and reconstituted as described previously (13).

### Crystallization and data collection of SkfB

SkfB was crystallized within an MBraun anaerobic chamber at 21 °C and under a N_2_ atmosphere using the sitting drop vapor diffusion method. Initial crystals of SkfB were obtained by sparse matrix screening using a mosquito pipetting robot (TTP LabTech). To obtain data-quality crystals, 1.5 μl of SkfB (10 mg/ml, 50 mm PIPES, pH 7.4, 150 mm KCl, 10 mm DTT, and 5 mm AdoMet) was mixed with 0.5 μl of precipitant (0.25 m magnesium formate and 15% PEG 3350) and equilibrated over a 500-μl reservoir. Brown crystals grew within 24 h and were transferred to a Coy Scientific anaerobic chamber (95% argon and 5% H_2_ atmosphere) for harvesting. To cryoprotect SkfB crystals, the crystals were transferred in four steps into a cryoprotectant solution containing 0.2 m magnesium formate, 20% PEG 3350, and glycerol. Upon each transfer, the glycerol concentration was increased to a final concentration of 20%. Crystals were then flash frozen in liquid nitrogen within the Coy chamber.

An anomalous data set and a native data set of SkfB were collected on the same crystal. The anomalous data set was collected to a resolution of 1.9 Å using an in-house CuK_α_ rotating anode source (Rigaku) with an imaging plate (RAXIS IV, Rigaku). To obtain phasing information, the data set was collected using a pseudo inverse beam in four 90° wedges with 1° increments. Following the home data collection, the crystal was shipped to the Advanced Photon Source (Argonne, IL) where a native data set was collected to a 1.29-Å resolution at beamline 24-ID-C using a Pilatus 6M pixel detector at a temperature of 100 K and a wavelength of 0.9792 Å. All data were processed in HKL 2000.

### Structure determination and refinement

The structure of SkfB with AdoMet was solved by an iron single-wavelength anomalous dispersion method in a *C*2 space group that contained one molecule in the asymmetric unit. Using anomalous data trimmed to 2.5-Å resolution, six iron sites (corresponding to one [4Fe-4S] cluster and one [2Fe-2S] cluster were identified in ShelxD (42) in HKL2MAP and refined in SHARP/autosharp (43, 44). The output experimental maps, with an initial figure of merit of 0.545–2.5-Å resolution, were of sufficient quality to trace the secondary structure of the AdoMet radical partial (β/α)_6_ TIM barrel domain and the C-terminal Twitch domain using BtrN (Protein Data Bank (PDB) code 4M7T) and MoaA (PDB code 1TV8) as guides. This initial model was used to define the solvent boundary during a subsequent round of solvent flattening in SOLOMON (45) and phase extension to 2.0-Å resolution. Loop regions and side chains with visible density were built into the resulting electron density.

When a near-completed model of SkfB was obtained, containing ∼350 of 410 residues, an AdoMet molecule, and the AdoMet radical cluster, the structure was refined against the full range of the native data set (50.0–1.29-Å resolution) using rigid-body atomic coordinates and atomic displacement (*B*-factor) refinements in Phenix (46). The resulting *R*_work_ and *R*_free_ were 24.4 and 29.5%, respectively. The model was completed by iterative rounds of model building in Coot (47) and refinement in Phenix (46). Waters were added manually in Coot during final rounds of refinement, and disordered side chains were truncated to the last atom with discernable density. The final model contained residues 12–322 and 330–401, one molecule of AdoMet, a [4Fe-4S] AdoMet radical cluster, and a [2Fe-2S] auxiliary cluster.

Composite omit maps calculated in Phenix (46) were used to validate the final structure, and MolProbity (48) was used to analyze the model geometry. MolProbity indicated that 98.15% of residues were found in the favored region, 1.85% in the allowed region, and 0.0% in the disallowed region of the Ramachandran plot, and ∼99% of residues have favorable rotamers. Figures were generated in PyMOL (49). Crystallography software packages were compiled by SBGrid (50).

### Expression, purification, and reconstitution of ^57^Fe-labeled SkfB for Mössbauer spectroscopy

SkfB variants containing ^57^Fe were prepared with slight adjustments to the previously published protocol (13). *Escherichia coli* Rosetta 2 (DE3) cells were cotransformed with pNB529 (SkfB with a tobacco etch virus–cleavable N-terminal His_6_ tag) and pDB1282 (plasmid containing *Azotobacter vinelandii* genes *iscS*, *iscU*, *iscA*, *hscA*, *hscB*, and *fdx* for iron-sulfur cluster biogenesis). A starter culture was incubated overnight in LB at 37 °C and 200 rpm. The next day, 15-ml aliquots of starter culture were pelleted by centrifugation (5000 × *g*) and gently resuspended in 15 ml of a modified M9 minimal medium containing 2% (w/v) glucose. Large-scale growth cultures containing M9 mineral medium (12 × 1.5 liters in 2.8-liter Fernbach flasks) supplemented with ampicillin (0.1 mg/ml), kanamycin (0.034 mg/ml), and ^57^FeSO_4_ (25 mm) were inoculated with 15 ml of resuspended starter culture cells. The large cultures were incubated at 37 °C and 175 rpm until the *A*_600_ nm reached 0.3, and arabinose and ^57^FeSO_4_ were added to final concentrations of 0.07% (w/v) and 0.05 mm, respectively, to induce pDB1282. After the addition of arabinose and ^57^FeSO_4_, the cultures were gradually cooled to 18 °C and slowed down to 150 rpm. After the cells reached an *A*_600_ nm of 0.6, expression from the *skfB* gene was induced upon addition of isopropyl β-d-1-thiogalactopyranoside to a final concentration of 0.1 mm. Cells were allowed to express SkfB at 18 °C for 18 h, harvested by centrifugation at 5000 × *g*, flash frozen in liquid nitrogen, and stored at −80 °C. SkfB and variant were purified and reconstituted as described previously (13) except that ^57^FeSO_4_ was used in the reconstitution.

### Mössbauer spectroscopy

Samples were prepared in a Coy Scientific anaerobic chamber. Sample buffer contained 50 mm PIPES, pH 7.4, 150 mm KCl, and 10 mm DTT. Concentrated protein was dispensed into Mössbauer cups and flash frozen with liquid nitrogen in the anaerobic chamber. With samples containing AdoMet, 10 molar eq of enzymatically generated AdoMet was incubated with concentrated SkfB for 20 min prior to flash freezing.

The ^57^Fe content of protein samples was determined by ICP-MS. SkfB was diluted to 1 μm (based on corrected Bradford concentration) in 1% (v/v) trace-meta-grade nitric acid. Analyses were conducted by the ICP-MS Metals Lab of the Department of Geology and Geophysics at the University of Utah.

Mössbauer spectra were measured on a spectrometer from WEB Research (Edina, MN) with an SVT-400 cryostat from Janis (Wilmington, MA) to maintain the temperature at 4.2 K. A 53-mT magnetic field was applied parallel to the propagation direction of the γ-beam. Isomer shifts are reported with respect to the centroid of the spectrum of α-iron metal at room temperature. Simulations of Mössbauer spectra were carried out with the program WMOSS (SEE Co., Edina, MN).

## Author contributions

T. A. J. G., W. M. K., N. A. B., E. J. B., C. K., V. B., and C. L. D. conceptualization; T. A. J. G., W. M. K., N. A. B., E. J. B., C. K., V. B., and C. L. D. investigation; T. A. J. G., V. B., and C. L. D. writing-original draft; T. A. J. G., W. M. K., N. A. B., C. K., V. B., and C. L. D. writing-review and editing; C. K., V. B., and C. L. D. funding acquisition; V. B. and C. L. D. supervision.

## Supplementary Material

Supporting Information

## Footnotes

5 The abbreviations used are:

sactipeptide: sulfur to α-carbon cross-linked peptide
RiPP: ribosomally synthesized and post-translationally modified peptide
AdoMet: *S*-adenosyl-l-methionine
SKF: sporulation killing factor
RRE: RiPP recognition element
5′-dAdo^•^: 5′-deoxyadenosyl radical
Aux: auxiliary iron-sulfur cluster
anSME: anaerobic sulfatase maturating enzyme
mT: millitesla(s)
ICP-MS: inductively coupled plasma mass spectrometry
TIM: triose phospahte isomerase.
