# Supplementary material for: Structural and spectroscopic analyses of the sporulation killing factor biosynthetic enzyme SkfB, a bacterial AdoMet radical sactisynthase
Source: J Biol Chem. 2018 Sep 14;293(45):17349–61. doi: 10.1074/jbc.RA118.005369 (PMC6231123; doi:10.1074/jbc.RA118.005369)
Supplement: Supporting Information [file supp_293_45_17349__index.html]

Structural and spectroscopic analyses of the sporulation killing factor biosynthetic enzyme SkfB, a bacterial AdoMet radical sactisynthase — Structural and Mössbauer characterization of SkfB — Structural and spectroscopic analyses of the sporulation killing factor biosynthetic enzyme SkfB, a bacterial AdoMet radical sactisynthase — Structural and Mössbauer characterization of SkfB — Supporting Information 

# Structural and spectroscopic analyses of the sporulation killing factor biosynthetic enzyme SkfB, a bacterial AdoMet radical sactisynthase

## Supporting Information

- Supporting Information (to be published online) - SI for the article.
