# Supplementary material for: Structural and spectroscopic analyses of the sporulation killing factor biosynthetic enzyme SkfB, a bacterial AdoMet radical sactisynthase
Source: J Biol Chem. 2018 Sep 14;293(45):17349–61. doi: 10.1074/jbc.RA118.005369 (PMC6231123; doi:10.1074/jbc.RA118.005369)
Supplement: Supporting Information [file supp_RA118.005369_140133_1_supp_200772_pbwt80.docx]

**Structural and spectroscopic analyses of the sporulation killing factor biosynthetic enzyme SkfB, a bacterial AdoMet radical sactisynthase**

Tsehai A.J. Grell^1^, William M. Kincannon^4^, Nathan A. Bruender^4#^, Elizabeth J. Blaesi^5^, Carsten Krebs^5,6*^, Vahe Bandarian^4*^, and Catherine L. Drennan^123*^

From the ^1^Department of Chemistry, ^2^Department of Biology, ^3^Howard Hughes Medical Institute, Massachusetts Institute of Technology, Cambridge, MA 02139, ^4^Department of Chemistry, University of Utah, Salt Lake City, UT 84112, ^5^Department of Chemistry, ^6^Department of Biochemistry and Molecular Biology, Penn State University, University Park, PA 16802.


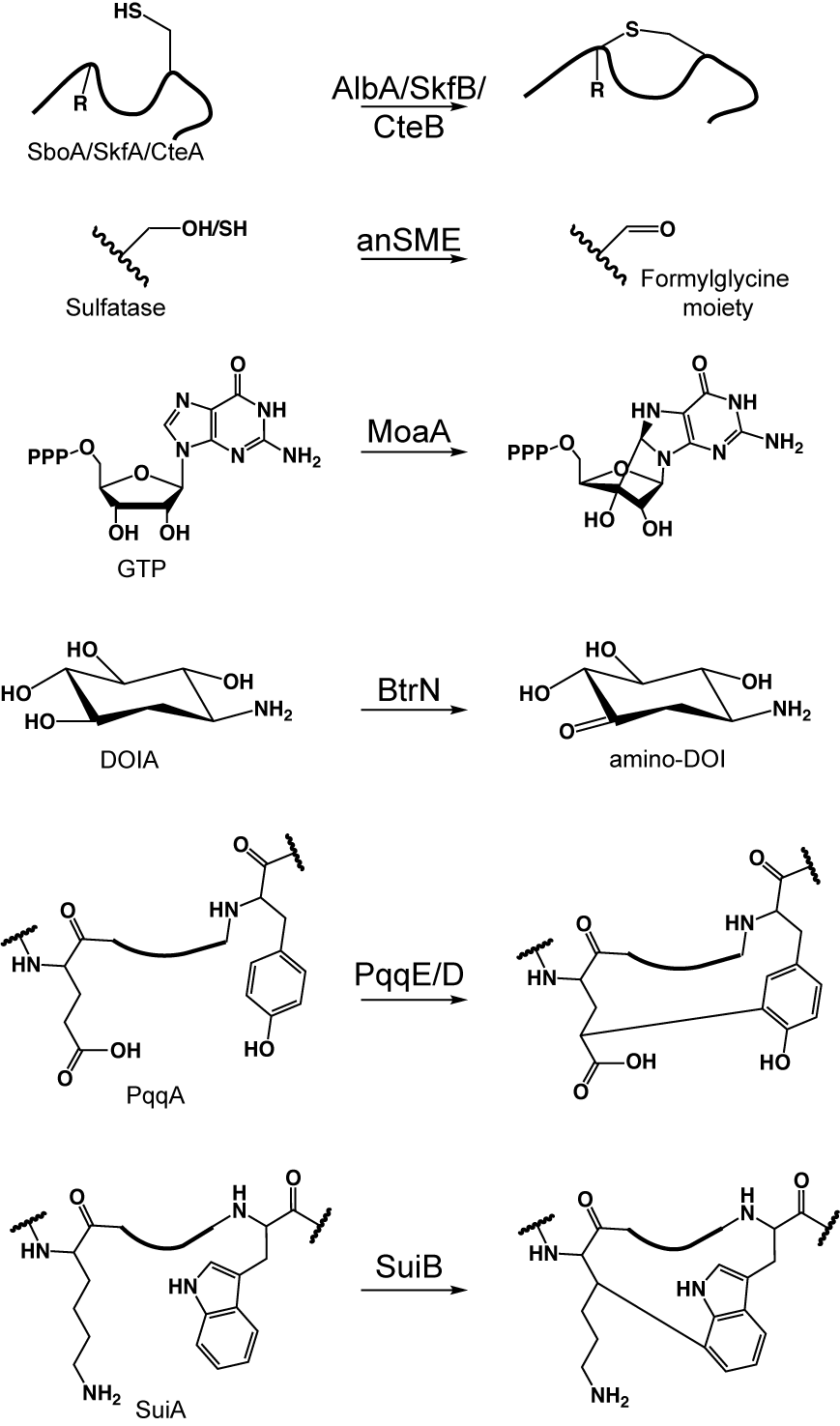


**Fig. S1. Reactions of SPASM/Twitch subclass members**. The sactisynthases AlbA, SkfB and CteB catalyze thioether bond (sactionine linkage) formation on their precursor peptides SboA, SkfA and CteA during the maturation of the sactipeptides subtilosin A, sporulation killing factor and thermocellin respectively. anSME installs the catalytically essential formylglycine in the active site of sulfatases. MoaA catalyzes the first step of molybdopterin biosynthesis; a complex rearrangement of guanosine triphosphate (GTP) and BtrN catalyzes the dehydrogenation of 2-deoxy-*scyllo*-inosamine (DOIA) during the biosynthesis of butirosin. In the first step of pyrroloquinoline quinone (PQQ) maturation, PqqE, in the presence of the peptide binding protein PqqD, forms a new carbon bond between glutamate and tyrosine residues of PqqA. Similarly, SuiB installs a lysine to tryptophan cross-link on SuiA in the biosynthesis of a streptide-like RiPP natural product. AlbA, CteB anSME, PqqE, and SuiB are SPASM enzymes whereas MoaA, BtrN, and SkfB are Twitch enzymes.

**
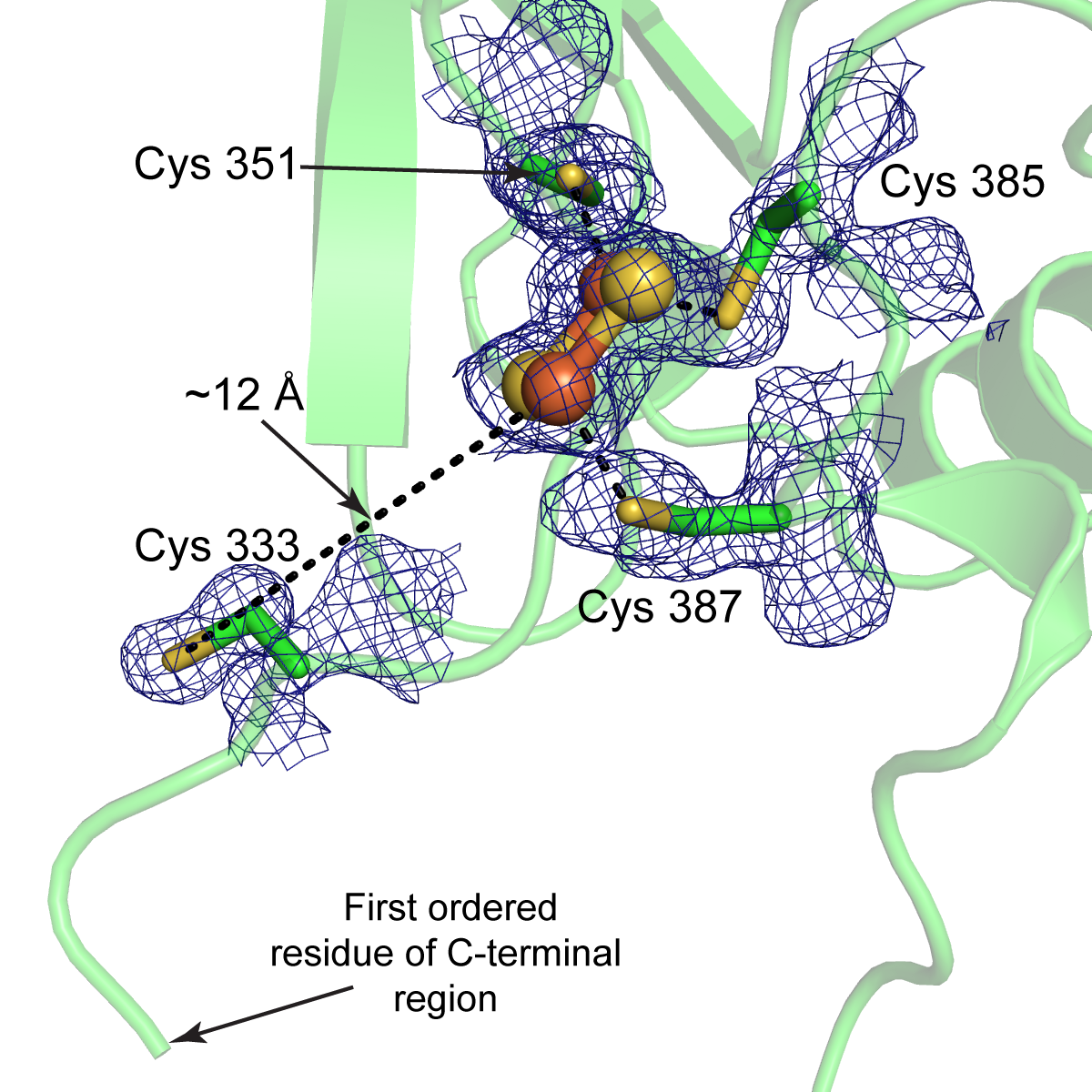
**

**Fig. S2. Location of the four expected cysteine cluster ligands to Aux I in SkfB.** Based on sequence analysis, four conserved cysteines are expected to coordinate Aux I of SkfB. Instead, the current structural data shows that the Aux I cluster-binding site is composed of three cysteine, C_351_, C_385_, and C_387_. The fourth cysteine, C_333_, is located ~12 Å away from the cluster and is one of the first ordered residues on the C-terminal end of the disordered linker between the AdoMet radical core domain and Twitch domain (residues 322-329 are disordered). A structure with an ordered linker would be useful to establish if C_333_ has an alternative position.





**Figure S3**. **Mössbauer spectra of SkfB.** 4.2-K/53-mT Mössbauer spectra of SkfB in the presence of the co-substrate AdoMet (black vertical bars) and absence of AdoMet (red line).

**

**

**Figure S4**. **Mössbauer spectra of the triple variant SkfB that is lacking cysteines ligands to AdoMet radical cluster.** Top: 4.2-K/53-mT Mössbauer spectrum of reconstituted SkfB triple variant. The spectrum can be simulated with parameters virtually identical to those associated with the [2Fe-2S]^2+^ cluster in wild-type SkfB (δ = 0.28 mm/s and ΔE_Q_ = 0.58 mm/s). Bottom: Comparison of the 4.2-K/53-mT Mössbauer spectrum of reconstituted wild-type SkfB (vertical bars) and that of the reconstituted triple variant of SkfB (solid line), scaled to 43 % of total intensity).

**Table S1**. Structurally characterized members of the SPASM/Twitch AdoMet radical subclass

| Enzyme | Description | C-terminal Domain | Aux I cluster identity | Substrates bound |
| --- | --- | --- | --- | --- |
| anSME | Sulfatase-maturating enzyme | SPASM | [4Fe-4S] cluster | AdoMet, residues 9-11 of a (18mer) sulfatase peptide mimic |
| MoaA | Molybdenum cofactor biosynthetic enzyme | Twitch | [4Fe-4S] cluster | AdoMet, GTP |
| BtrN | Butirosin biosynthetic enzyme | Twitch | [4Fe-4S] cluster | AdoMet, 2-deoxy-*scyllo* inosamine (DOIA) |
| CteB | Sactipeptide maturating enzyme | SPASM | [4Fe-4S] cluster | AdoMet, residues -18 to -10 and 2 to 3 of a (18mer) N-terminal CteA peptide |
| PqqE | Pyrroloquinoline quinone biosynthetic enzyme | SPASM | [2Fe-2S] cluster | none |
| SuiB | Streptide biosynthetic enzyme | SPASM | [4Fe-4S] cluster | AdoMet, residues -13 to -1 of the precursor peptide of SuiA |
| SkfB | Twitch sactipeptide maturating enzyme | Twitch | [2Fe-2S] cluster | AdoMet |

**Table S2.** Primers used for all cloning described in the Experimental Procedures.

| **Primer** | **Sequence** |
| --- | --- |
| C117A  Forward | CCTTACAGTTGACAAATGCTGCTAATTTAAGCTGTTCGTTTTGC |
| C117A  Reverse | GCAAAACGAACAGCTTAAATTAGCAGCATTTGTCAACTGTAAGG |
| C117A/C121A/C124A  Forward | CCTTACAGTTGACAAATGCTGCTAATTTAAGCGCT  TCGTTTGCCTATGCCAGCTCAGGTAAACCATATCC |
| C117A/C121A/C124A  Reverse | GGATATGGTTTACCTGAGCTGGCATAGGCAAACGA  AGCGCTTAAATTAGCAGCATTTGTCAACTGTAAGG |
